# Prevalence and Predictors of *Toxoplasma gondii* Infection in Psychiatric Inpatients in Fars Province, Southern Iran

**DOI:** 10.3389/fpsyt.2022.891603

**Published:** 2022-06-14

**Authors:** Aref Teimouri, Othman Jamal Nassrullah, Pouya Hedayati, Mohammad Saleh Bahreini, Rasoul Alimi, Sina Mohtasebi, Amir Masoud Salemi, Qasem Asgari

**Affiliations:** ^1^Department of Parasitology and Mycology, School of Medicine, Shiraz University of Medical Sciences, Shiraz, Iran; ^2^Department of Clinic and Medicine, College of Veterinary Medicine, University of Sulaimani, Sulaymaniyah, Iraq; ^3^Department of Epidemiology and Biostatistics, School of Health, Torbat Heydariyeh University of Medical Sciences, Torbat Heydariyeh, Iran; ^4^Department of Medical Parasitology and Mycology, School of Public Health, Tehran University of Medical Sciences, Tehran, Iran

**Keywords:** psychiatric patients, toxoplasmosis, risk factors, suicide attempts, Iran

## Abstract

**Background:**

Psychiatric patients are at increased risk of exposure to *Toxoplasma gondii* infection, which may be linked to their living facilities and behaviors. Limited knowledge on the prevalence of *T. gondii* infection and its associated risk factors in psychiatric patients are available to the international medical communities. Thus, the aim of the current study was to assess seroprevalence of *T. gondii* and its associated risk factors in psychiatric inpatients in Fars Province, southern Iran.

**Methods:**

This cross-sectional study was carried out on psychiatric patients hospitalized in Ibn Sina Hospital affiliated to Shiraz University of Medical Sciences, Fars Province, southern Iran, March to July 2021. Blood samples were collected from 318 psychiatric patients and assessed for the detection of IgG against *T. gondii* using enzyme-linked immunosorbent assay (ELISA). Moreover, structured questionnaires were completed for the participants at the time of sampling. Logistic regression analysis was used to assess possible associations between the latent toxoplasmosis and the variables.

**Results:**

The overall seroprevalence of anti-*T. gondii* IgG in psychiatric inpatients was 22.3% (71/318; 95% CI = 17.9–27.3). Multivariate analyses revealed that age > 30 years [adjusted odds ratio (AOR) = 2.24, 95% CI = 1.10–4.60, *p* = 0.03], contact with cats (AOR = 2.52, 95% CI = 1.14–5.58, *p* = 0.03), raw vegetable consumption (AOR = 3.65, 95% CI = 1.74–7.65, *p* = 0.001), raw/undercooked meat consumption (AOR = 4.30, 95% CI = 1.47–12.63, *p* = 0.008), suicide attempt (AOR = 3.77, 95% CI = 1.58–8.97, *p* = 0.003) and cigarette smoking history (AOR = 0.38, 95% CI = 0.17–0.83, *p* = 0.02) were independent risk factors for *T. gondii* infection.

**Conclusion:**

The current results demonstrated that contact with cats, raw vegetable consumption and raw/undercooked meat consumption were independent risk factors for *T. gondii* seropositivity. Moreover, the current study showed significant associations between seropositivity of *T. gondii* and suicide attempts as well as negative associations between seropositivity of *T. gondii* and cigarette smoking in psychiatric inpatients using multivariate logistic regression.

## Introduction

*Toxoplasma gondii* (*T. gondii*), the causative agent of toxoplasmosis, is a ubiquitous parasite of warm-blooded animals and is one of the most common parasitic protozoans of humans worldwide. Humans majorly become infected with the parasite *via* consumption of raw and/or undercooked meats containing tissue cysts or accidental consumption of food and water contaminated with oocysts excreted in cat feces. Although toxoplasmosis is generally asymptomatic in immunocompetent individuals, disease can be complicated and life-threatening in such patients, including organ transplant recipients, cancer patients and those with human immunodeficiency virus (AIDS) (1–3). Primary *T. gondii* infection in pregnant women can cause severe damages to the fetus and may result in irreversible conditions such as abortion as well as severe neurodevelopmental malformations such as hydrocephaly and microcephaly (4, 5). Latent toxoplasmosis is frequently associated to tissue cysts of *T. gondii* in the skeletal muscles and brain tissues, leading to increased risks of psychiatric and neurological abnormalities and personality changes (6). It has been well documented that toxoplasmosis may lead to changes in behaviors of humans (7). For example, recent clinical studies have demonstrated that antibodies against *T. gondii* infection may play roles in pathophysiology of suicide (8). Studies have revealed that increased levels of cytokines are associated to depression and suicide (9).

In Iran, toxoplasmosis has become a serious infection since the first epidemic investigation of the disease in humans was carried out in Caspian Sea region (1978) using indirect fluorescent antibody (IFA) technique (10). Although several studies have been carried out on the seroprevalence of *T. gondii* infection in Iran, most of these studies have focused on high risk populations, including AIDS and cancer patients as well as pregnant and premarital women (11–14). To date, several studies have been carried out on the seroprevalence of toxoplasmosis in psychiatric inpatients. However, most of these studies have merely been carried out to assess the seroprevalence of *T. gondii* infection and have not evaluated the possible associated risk factors and contamination routes of *T. gondii* infection (15). Generally, people with mental illnesses are at increased risk of exposure to *T. gondii* infection, which may be linked to their living facilities and behaviors. Furthermore, these group of people are generally at increased risks of medical comorbidities due to their decreased resistance to infections. This decreased resistance may occur because of age, underlying medical problems and drug abuse (16–18).

Usually, several factors affect prevalence of toxoplasmosis, including sex, age, residence, education background, and dietary habits (19). Although, seroprevalence of *T. gondii* antibodies in general adult population in Iran has been 39.3% (95% CI = 33.0–45.7), ranging 12–87.5% with further endemicity in northern provinces (20), limited knowledge are available on the prevalence of *T. gondii* infection and its associated risk factors in psychiatric patients. Therefore, the current study was carried out to assess seroprevalence of *T. gondii* infection and identify its associated risk factors as well as assessing possible contamination routes of toxoplasmosis in a population of psychiatric inpatients in Fars Province, southern Iran.

## Materials and Methods

### Study Design and Participants

This cross-sectional study was carried out on psychiatric patients hospitalized in Ibn Sina Hospital affiliated to Shiraz University of Medical Sciences, Fars Province, southern Iran, March to July 2021. Psychiatric diagnosis included real-world clinical diagnosis by two experienced neuropsychiatrists and neuropsychologists, according to the fifth version of the Guidelines for Diagnosis and Statistics of Mental Disorders (DSM-V) which is routinely used in Iranian psychiatric practices. Psychiatric patients were classified into four major groups based on DSM-V and clinical criteria (21), including anxiety, mood, personality, and psychotic disorders. Anxiety disorders group including panic, obsessive-compulsive, social and specific phobia, posttraumatic stress, generalized anxiety, and posttraumatic stress disorders. Mood disorders group including major depressive, dysthymic, bipolar, and related disorder due to another medical condition, cyclothymic, and substance/medication-induced depressive disorder. Personality disorders group including paranoid, schizoid, schizotypal, borderline, antisocial, histrionic, narcissistic, avoidant, dependent, and obsessive-compulsive personality disorders. Psychotic disorders group including schizophrenia, schizophreniform, schizoaffective, and delusional disorders, as well as substance/medication-induced psychotic disorder, and psychotic disorder due to another medical condition. Inclusion criteria for the participants were being psychiatric inpatient, aging 15 years and older, having full consent to participate in the study and being resident of Fars Province. The exclusion criteria were being non-Iranian citizen and traveler. In total, 16 patients were excluded from the study because they did not accept to participate or fill the questionnaire. Sample size was calculated based on the prevalence of toxoplasmosis in the region using standard statistical formula for the estimation of sample sizes for a proportion with a disease prevalence rate of ∼25% (22), margin of error of 0.05 and 95% confidence interval (CI), resulting in a sample size of 289. To include the non-response rate, sample size was inflated by 10% to create a total sample size of 318.

### Serum Collection and Assessment

Samples included 318 sera from psychiatric patients hospitalized in Ibn Sina Hospital. A venous blood sample (up to 3 mL) was collected from each participant and sera were separated by centrifugation at 4,000 rpm for 5 min. Sera were stored at –20^°^C until use. Specific anti-*T. gondii* IgG levels were assessed using commercially available enzyme-linked immunosorbent assay (ELISA) kit (Vircell, Granada, Spain) with a diagnostic sensitivity of > 98% (95% CI: 88–100) and specificity of 100% (95% CI: 89–100) according to the manufacturer’s instructions. Results were interpreted using the manufacturer’s recommendations as follows: sera with antibody index < 9 were reported as negative, 9–11 as equivocal and > 11 as positive sera (23). Laboratory tests were performed blindly, so that the specialist technician who tested the samples did not know the health status of the people whose serum samples were tested.

### Questionnaire

A questionnaire was completed for each participant. Demographic information of the questionnaire included sex, age, residency, education background, and possible risk factors included having cats as pets, contact with cats, raw and/or unwashed vegetable consumption, drinking unsanitary water, raw/undercooked meat consumption, gardening or agricultural activities and blood transfusion. Moreover, further information such as duration of mental illness, suicide attempt, cigarette smoking history and miscarriage history in women were collected at the time of sampling using questionnaires.

### Statistical Analysis

Statistical Package for the Social Sciences (SPSS) Software v.21.0 (IBM, United States) was used for the analysis of data (24). Data were described using calculation of frequencies (%) and 95% CI. To analyze associations between *T. gondii* infection and potential risk factors, logistic regression was used followed by multivariate logistic analysis with the full model, including all potential risk factors in analyses to control effects of potential confounding factors. Strength of the associations between the predictors and outcome variables was assessed using adjusted odds ratio (AOR) and 95% CI. Overall number of the participants included in the unadjusted and adjusted regression models varied based on the completeness of the putative confounding variables and all missing data were excluded from the regression modeling. However, proportion of the missing data was relatively small, ranging 0–0.9%. In general, *p*-values less than 0.05 were reported statistically significant.

## Results

### General Characteristics of the Participants

Results of sociodemographic characteristics and risk factors linked to seroprevalence of *T. gondii* in 318 psychiatric inpatients are shown in Table 1. The mean age of the participants was 35.91 y ± 12.23 with minimum and maximum ages of 15 and 76 years, respectively. Of 318 participants, 183 (57.5%, 95% CI: 51.9–60.3) were males (mean age 35.96 ± 12.80) and 135 (42.5%, 95% CI: 37.0–48.1) were females (mean age 35.84 ± 11.48); differences in ages between the two sexes were not significant (*t* = 0.086, *p* = 0.93). A majority of the participants were residents of the city (*n* = 228, 71.7%). Out of 318 psychiatric patients, almost half (*n* = 144, 45.3%) had mood disorders followed by psychotic disorders (*n* = 81, 25.5%) and anxiety disorders (*n* = 68, 21.3%), whereas personality disorders were the least disorders (*n* = 25, 7.8%) (Table 1).

**TABLE 1:** Demographic characteristics and risk factors linked to seroprevalence of *T. gondii* in 318 psychiatric patients.

Characteristics	Frequency	Percent (95% CI)
Age		
≤ 30 years. > 30 years.	139 179	43.7 (38.2–49.4) 56.3 (50.6–61.8)

Gender		
Male Female	183 135	57.5 (51.9–60.3) 42.5 (37.0–48.1)

Level of education		
Primary (grades 1–5) Secondary (grades 6–9) High school (grades 10–12) University	33 31 111 143	10.4 (7.3–14.3) 9.7 (6.7–13.6) 34.9 (29.7–40.4) 45.0 (39.4–50.6)

Father’s level of education		
Primary (grades 1–5) Secondary (grades 6–9) High school (grades 10–12) University	117 14 92 95	36.8 (31.5–42.4) 33.3 (28.2–38.8) 28.9 (24.0–34.3) 29.9 (24.9–35.2)

Mental disorder		
Anxiety Mood Personality Psychotic	68 144 25 81	21.3 (17.0–26.3) 45.3 (39.7–50.9) 7.9 (5.2–11.4) 25.5 (20.8–30.6)

Residency		
City Rural	228 90	71.7 (66.4–76.6) 28.3 (23.4–33.6)

Cat ownership		
Yes No	28 290	8.8 (5.9–12.5) 91.2 (87.5–94.1)

Contact with cats		
Yes No	71 247	22.3 (17.9–27.3) 77.7 (72.7–82.1)

Raw vegetable consumption		
Yes No	73 245	23.0 (18.4–28.0) 77.0 (72.0–81.6)

Gardening or agricultural activities		
Yes No	53 265	16.7 (12.7–21.2) 83.3 (78.8–87.3)

Unsanitary water drinking		
Yes No	54 264	17.0 (13.0–21.6) 83.0 (78.4–87.0)

Raw/undercooked meat consumption		
Yes No	24 294	7.5 (4.9–11.0) 92.5 (89.0–95.1)

Blood transfusion		
Yes No	8 310	2.5 (1.1–4.9) 97.5 (95.1–98.9)

Suicide attempts		
Yes No	38 280	11.9 (8.6–16.0) 88.1 (84.0–91.4)

Duration of mental illness (missing values *n* = 3)		
≤3 years >3 years	171 144	54.3 (48.6–59.9) 45.7 (40.1–51.4)
History of miscarriage (missing values *n* = 1)		
Yes No	25 109	18.7 (12.5–26.3) 81.3 (73.7–87.5)

Cigarette smoking history		
Yes No	114 204	35.8 (30.6–41.4) 64.2 (58.6–69.4)

Total	318	100

### Seroprevalence and Associated Risk Factors of *Toxoplasma gondii* in Psychiatric Inpatients

The overall seroprevalence of anti-*T. gondii* IgG in psychiatric patients was 22.3% (71/318; 95% CI = 17.9–27.3). In univariate analysis, several variables were significantly associated to seropositivity of *T. gondii*, including age > 30 years [crude odds ratio (COR) = 2.83, 95% CI = 1.57–5.10, *p* = 0.001], female sex (COR = 1.79, 95% CI = 1.04–3.03, *p* = 0.03), living in rural areas (COR = 3.14, 95% CI = 1.80–5.44, *p* < 0.001), having cats in the household (COR = 2.94, 95% CI = 1.32–6.54, *p* = 0.008), contact with cats (COR = 2.83, 95% CI = 1.59–5.05, *p* < 0.001), raw vegetable consumption (COR = 4.90, 95% CI = 2.75–8.73, *p* < 0.001), gardening or agricultural activities (COR = 2.29, 95% CI = 1.21–4.33, *p* = 0.01), raw/undercooked meat consumption (COR = 5.82, 95% CI = 2.46–13.78, *p* < 0.001), suicide attempt (COR = 3.39, 95% CI = 1.67–6.86, *p* = 0.001), duration of mental illness > 3 years (COR = 1.81, 95% CI = 1.06–3.09, *p* = 0.03) and cigarette smoking history (COR = 0.54, 95% CI = 0.30–0.97, *p* = 0.04) (Table 2).

**TABLE 2:** Logistic regression analysis of the risk factors associated to anti-*T. gondii* IgG in 318 psychiatric patients based on univariate and multivariate analyses.

Group	Anti-*T. gondii* IgG		Univariate analysis	Multivariate analysis
	Negative no. (%)	Positive no. (%)	COR (95% CI)	AOR (95% CI)
Age				
≤30 years >30 years	121 (87.1) 126 (70.4)	18 (12.9) 53 (29.6)	1 2.83 (1.57–5.10)**	1 2.24 (1.10–4.60)*

Sex				
Male Female	150 (82.0) 97 (71.9)	33 (18.0) 38 (28.1)	1 1.79 (1.04–3.03)*	1 1.13 (0.55–2.29)

Level of education				
Primary (grades 1–5) Secondary (grades 6–9) High school (grades 10–12) University	25 (75.8) 21 (67.7) 89 (80.2) 112 (78.3)	8 (24.2) 10 (32.3) 22 (19.8) 31 (21.7)	1.16 (0.48–2.82) 1.72 (0.73–4.03) 0.89 (0.48–1.65) 1	– – –

Father’s level of education				
Primary (grades 1–5) Secondary (grades 6–9) High school (grades 10–12) University	92 (78.6) 9 (64.3) 74 (80.4) 72 (75.8)	25 (21.4) 5 (35.7) 18 (19.6) 23 (24.2)	0.85 (0.45–1.62) 1.74 (0.53–5.72) 0.76 (0.38–1.53) 1	– – –

Mental disorder				
Anxiety Mood Personality Psychotic	53 (77.9) 116 (80.6) 18 (72.0) 60 (74.1)	15 (22.1) 28 (19.4) 7 (28.0) 21 (25.9)	1 0.85 (0.42–1.73) 1.37 (0.48–3.91) 1.24 (0.58–2.64)	– – – –

Residency				
City Rural	191 (83.8) 56 (62.2)	37 (16.2) 34 (37.8)	1 3.14 (1.80–5.44)***	1 1.72 (0.86–3.44)

Cat ownership				
Yes No	16 (57.1) 231 (79.7)	12 (42.9) 59 (20.3)	2.94 (1.32–6.54)* 1	1.20 (0.38–3.83) 1

Contact with cats				
Yes No	44 (62.0) 203 (82.2)	27 (38.0) 44 (17.8)	2.83 (1.59–5.05)*** 1	2.52 (1.14–5.58)* 1

Raw vegetable consumption				
Yes No	39 (53.4) 208 (84.9)	34 (46.6) 37 (15.1)	4.90 (2.75–8.73)*** 1	3.65 (1.74–7.65)** 1

Gardening or agricultural activities				
Yes No	34 (64.2) 213 (80.4)	19 (35.8) 52 (19.6)	2.29 (1.21–4.33)* 1	1.22 (0.54–2.78) 1

Unsanitary water drinking				
Yes No	39 (72.2) 208 (78.8)	15 (27.8) 56 (21.2)	1.43 (0.74–2.78) 1	– –

Raw/undercooked meat consumption				
Yes No	10 (41.7) 237 (80.6)	14 (58.3) 57 (19.4)	5.82 (2.46–13.78)*** 1	4.30 (1.47–12.63)** 1

Blood transfusion				
Yes No	7 (87.5) 240 (77.4)	1 (12.5) 70 (22.6)	0.49 (0.06–4.05) 1	– –

Suicide attempts				
Yes No	21 (56.3) 226 (80.7)	17 (44.7) 54 (19.3)	3.39 (1.67–6.86)** 1	3.77 (1.58–8.97)** 1

				
Duration of mental illness (missing values *n* = 3)				
≤ 3 years > 3 years	141 (82.5) 104 (72.2)	30 (17.5) 40 (27.8)	1 1.81 (1.06–3.09)*	1 1.86 (0.96–3.60)

History of miscarriage (missing values *n* = 1)				
Yes No	81 (74.3) 15 (60.0)	28 (25.7) 10 (40.0)	1.93 (0.78–4.78) 1	– –

Cigarette smoking history				
Yes No	96 (84.2) 151 (74.0)	18 (15.8) 53 (26.0)	0.54 (0.30–0.97)* 1	0.38 (0.17–0.83)* 1

*Significance at < 0.05; **significance at < 0.01; ***significance at < 0.001. CI, confidence interval; COR, crude odds ratio; AOR, adjusted odds ratio.

Multivariate analysis revealed that age > 30 years [adjusted odds ratio (AOR) = 2.24, 95% CI = 1.10–4.60, *p* = 0.03], contact with cats (AOR = 2.52, 95% CI = 1.14–5.58, *p* = 0.03), raw vegetable consumption (AOR = 3.65, 95% CI = 1.74–7.65, *p* = 0.001), raw/undercooked meat consumption (AOR = 4.30, 95% CI = 1.47–12.63, *p* = 0.008), suicide attempt (AOR = 3.77, 95% CI = 1.58–8.97, *p* = 0.003), and cigarette smoking history (AOR = 0.38, 95% CI = 0.17–0.83, *p* = 0.02) were independent risk factors for *T. gondii* infection (Table 2). Results of the association between suicide attempts and *T. gondii* seropositivity in 318 psychiatric patients are shown in Table 3. *T. gondii* seropositivity was significantly associated with suicide attempts in anxiety and psychotic disorder groups (Table 3).

**TABLE 3:** Association between suicide attempts and *T. gondii* seropositivity in 318 psychiatric patients.

Mental disorder	Suicide attempts	Anti-*T. gondii* IgG		*p*-value
		Negative no. (%)	Positive no. (%)	
Anxiety				
	Yes No	0 (0.0) 53 (100.0)	2 (13.3) 13 (86.7)	0.046*
Mood				
	Yes No	19 (16.4) 97 (83.6)	9 (32.1) 19 (67.9)	0.067
Personality				
	Yes No	– 18 (100.0)	– 7 (100.0)	–
Psychotic				
	Yes No	2 (3.3) 58 (96.7)	6 (28.6) 15 (71.4)	0.003*

*Significance at < 0.05.

## Discussion

In this study, the overall seroprevalence of *T. gondii* infection in psychiatric patients was 22.3%, which was approximately similar to the seroprevalence rate of *T. gondii* infection in previous studies on the general population and students of Fars Province with an overall prevalence of 21.3% (22) and 27.6% (25), respectively. However, the seroprevalence rate of *T. gondii* infection in the present study was lower than that in studies on psychiatric patients in Nigeria (32.1%) (26), northwest Ethiopia (33.6%) (27), Libya (41.7%) (28), and western Romania (57.7%) (29). In contrast, prevalence of *T. gondii* infection in psychiatric patients of the current study was higher than that in studies in eastern China (17.3%) (30) and northern Mexico (18.2%) (31). A possible explanation for the differences might be linked to local cultures, dietary habits, socioeconomic statuses, geographic regions, personal sanitary/hygiene levels, types of the laboratory methods, environmental exposures and spreads of cats in the regions (32).

Results of the present study have shown that *T. gondii* infection is significantly associated to the age of psychiatric patients. The OR of *T. gondii* infection increased up to 2.24 (CI = 1.10–4.60, *p* = 0.03) in participants aged > 30 years, compared to that in participants aged ≤ 30 years. Results were similar to those of previous surveys (33, 34). In a study by Fan et al. (35), a significantly higher seroprevalence (80%) was reported in the age group of ≥ 45 years (35). In a survey by Yang et al. (36), seroprevalence of *T. gondii* infection showed upward trends with aging and the seroprevalence rate in postgraduate students (2.46%) (≥ 22 years old) was higher than that in undergraduates (1.63%) (≤ 21 years old) (36). Further outdoor activities and longer exposures to the risk factors of infectious sources include possible explanations for further seroprevalences in older people (37, 38). However, in previous studies, *T. gondii* infection in psychiatric patients was not significantly affected by age (30). This might be resulted from the broad differences in living conditions within the countries as well as lifestyles and study periods.

In the current study, multivariate analysis showed that individuals who contacted with cats and consumed raw vegetables and raw/undercooked meats were, respectively, 2.52, 3.65, and 4.30 times more likely to become infected with *T. gondii* than those who did not contact with cats and consume raw vegetables and raw/undercooked meats. Constant shedding of oocysts from the infected cats, as the main reservoirs of *T. gondii*, plays a key role in infection of humans. Moreover, contact with cats has been reported as a risk factor in previous seroepidemiologic studies (39–41). Cats are commonly held as pets in Iran. However, their potential roles in contamination of the environment with *T. gondii* are neglected. Studies have verified that consumption of infected raw/undercooked meats or infective oocytes *via* raw vegetables and fruits are independent risk factors in human infection with *T. gondii* (42–46). It is noteworthy to increase public information of preventive methods against toxoplasmosis with particular emphases on the critical roles of felines in the spread of *T. gondii* as well as associations between *T. gondii* infection and behavioral characteristics.

Well-known factors of other studies associated to *T. gondii* infection, including contact with garden soil (47) and residency in a community (36), were not associated to *T. gondii* infection in the current study. This is in contrast to previous studies carried out in Gondar (48) and Bench Maji (49) regions of Ethiopia. Yang et al. (36) reported that living in rural areas and gardening or agriculture were independent risk factors for *T. gondii* seropositivity using multivariate logistic regression analysis; in contrast to the current study (36). In a recent study by Achaw et al. (27), farming (gardening and agriculture) (AOR = 2.058, CI = 1.018–4.163, *p* = 0.045) was significantly associated to the seroprevalence of anti-*T. gondii* IgG (27). It could be resulted from the significant survivals of oocysts, remaining infective for up to 24 m under favorable conditions. This could be a potential risk factor for the increased seroprevalence of anti-*T. gondii* IgG (50). Fars province is located in the south of Iran and has a dry climate with hot sunny summers and relatively cold winters. Environmental conditions and regional climate affect the survival of *T. gondii* oocysts. Rainfalls, tipping the balance in favor of the survival of the oocysts in the soil. It has been well documented that infection is more prevalent in humid tropical areas than in cold climates and arid areas (19, 20). However, the seroprevalence of human toxoplasmosis depends on several anthropogenic factors such as dietary habits and hygiene practices (19).

In the present study, significant associations were detected between seropositivity to *T. gondii* and suicide attempt in psychiatric inpatients. This suggests that *T. gondii* infection increases the risk of suicide with an OR of 3.77. Similar to the present study, several studies have shown that *T. gondii* infection represents a risk factor for suicidal behaviors (51). Associations of *T. gondii* seropositivity with suicide attempt, recurrent mood disorders and psychiatric patients have been documented in South Korea (52), United States (8), and Mexico (53). More recently, associations between *T. gondii* seropositivity and suicidal behaviors have been significant in Mexican patients attending primary health care clinics and those with alcohol consumption (54). In a study by Zhang et al., associations between *T. gondii* infection and non-fatal suicidal self-directed violence showed that *T. gondii* infected individuals had 7.12 times more significant risk of non-fatal suicide attempts (55). Ling et al. reported significant upward trends in seropositivity of *T. gondii* and risks of suicide in women older than 45 years (56). Furthermore, numerous studies on European and Chinese nations highlighted that countries with higher *T. gondii* prevalence rates included higher suicide rates (57). Interestingly, a case study reported that depressive symptoms were completely improved following treatment of *T. gondii* infection; however, antidepressant therapy did not resolve patients’ depressive symptoms (58). A recent systematic review and meta-analysis demonstrated that odds of suicide in individuals with *T. gondii* seropositivity was 43% (OR: 1.43, 95% CI; 1.15–1.78) higher than that in individuals with no infection (51).

Pathophysiological mechanisms of *T. gondii* in suicide behaviors have not been discovered clearly. Within numerous theories describing possible relationships between *T. gondii* infection and suicide behavior, direct effects of *T. gondii* on neuronal function and immune-mediated dopamine and serotonin syntheses is a highlighted hypothesis. In this scenario, host immune response against *T. gondii* infection produces pro-inflammatory cytokines (IFN- γ, IL-6, and IL-12) through activation of Th cells and macrophages (59). The produced IFN- γ blocks development of *T. gondii* by inducing activation of enzymes of kynurenine monooxygenase (KMO) and indoleamine 2,3-dioxygenase (IDO), resulting in tryptophan depletion (60, 61). Resultant tryptophan depletion results in decreases in serotonin production in the brain, which may increase human susceptibility to trigger suicide risk factors such as depression, impulsivity and aggression (62). Moreover, the activation of kynurenine pathway in itself as well as the imbalance between neurotoxic and neuroprotective metabolites could trigger the depression (63). The highlighted inflammatory pathways and associated chemical compounds affecting the nervous system can play key roles in behavioral development that triggers suicide attempts (64, 65). In contrast, the current results deny associations between *T. gondii* infection and suicidal attempts discovered in a study on patients suffering from mental and behavioral disorders due to psychoactive substance use in Mexico (66), adolescents in Turkey (67) and general population study in Finland (68). Further studies with a wide range of sociodemographic, clinical and behavioral variables are necessary to investigate associations between *T. gondii* infection and suicide attempts.

Another finding of the present study included that *T. gondii* infection in psychiatric patients was negatively associated to smoking habits (AOR = 0.38, 95% CI = 0.17–0.83, *p* = 0.02). Infection with *T. gondii* and smoking are linked to dopamine release. Naturally, dopamine signals pleasure and is considered a mood booster. This pleasure reaction and glee resulting from dopamine is a significant part of nicotine and smoking addictions (69). Regarding possible mechanisms involved in this process, *T. gondii* seropositivity might affect key dopaminergic pathways of the nervous system and decline the desire or motivation of smoking. Chronic *T. gondii* infection usually causes specific changes in chemical messengers used by inter-neuronal connections of the brain. Decoding genome of *T. gondii* has revealed that amino acid hydroxylase, a rate-limiting enzyme in dopamine synthesis, is coded in two loci with high homologies for mammalian genes (70). These homologies demonstrate that the presence of *T. gondii* in the brain can increase availability of dopamine, leading to the feeling of pleasure and satisfaction (71, 72). Furthermore, experimental studies have verified that *T. gondii* infection triggers extra releases of dopamine in the brain of infected mice (73). Similarly, pharmaceutical compounds interfering with dopamine metabolism prevent effects of *T. gondii* on mental manners (74). Associations of tobacco addiction with detrimental health outcomes such as hepatitis C virus (75), postoperative infections after dental implants (76) and spine surgeries (77) have been well-documented. However, mechanisms underlying associations of the infection with tobacco smoking are poorly described. In terms of the limitations, save for the DSM-V guideline, no independent tools were administrated to confirm the clinical diagnosis of patients, and also, it should be noted that IgG assessment in a single serum sample, cannot estimate the exact time of acquiring the *T. gondii* infection.

## Conclusion

The present study showed that the overall seroprevalence of *T. gondii* infection in psychiatric patients was 22.3%. Multivariate analyses demonstrated that contact with cats, raw vegetable consumption, and raw/undercooked meat consumption were independent risk factors for *T. gondii* seropositivity. Moreover, the current study revealed significant associations between seropositivity of *T. gondii* and suicide attempts as well as negative associations between seropositivity of *T. gondii* and cigarette smoking in psychiatric inpatients using multivariate logistic regression.

## Data Availability Statement

Data generated in this study are included in the published article. Data analyzed during the current study are publicly available via Figshare Repository, https://figshare.com/s/111fbbe17cbb6b6c62f3.

## Ethics Statement

The studies involving human participants were reviewed and approved by the research Ethics Committee of Shiraz University of Medical Sciences. All participants agreed that their participation was voluntary and was informed that the methodology included no potential risks and that their information was assumed strictly confidential. This study was approved by the research Ethics Committee of Shiraz University of Medical Sciences with an ethical code: IR.SUMS.MED.REC.1399.580. Written informed consent to participate in this study was provided by the participants’ legal guardian/next of kin.

## Author Contributions

AT and QA conceived and designed the study. PH collected the sample from the hospital. MB, AS, and PH contributed in ELISA method. AT, OJN, SM, RA, and QA analyzed and interpreted the data. AT and OJN prepared the original draft manuscript. AT and SM contributed to review and editing of the final version of manuscript. All authors have read and approved the final manuscript.

## Conflict of Interest

The authors declare that the research was conducted in the absence of any commercial or financial relationships that could be construed as a potential conflict of interest.

## Publisher’s Note

All claims expressed in this article are solely those of the authors and do not necessarily represent those of their affiliated organizations, or those of the publisher, the editors and the reviewers. Any product that may be evaluated in this article, or claim that may be made by its manufacturer, is not guaranteed or endorsed by the publisher.
